# Yet another modification of Guedel’s airway

**DOI:** 10.4103/0019-5049.76579

**Published:** 2011

**Authors:** Vanita Ahuja, Virender K Arya, Babloo Kumar

**Affiliations:** Department of Anaesthesia & Intensive Care, Government Medical College and Hospital, Sector-32, Chandigarh, India; 1 Department of Anaesthesia & Intensive Care, Postgraduate Institute of Medical Education and Research, Chandigarh, India

Sir,

Flexible fibreoptic laryngoscopic intubation (FFLI) is a very reliable approach to difficult airway management.[1] Oral FFLI is considered more difficult than nasal FFLI as the tip enters the larynx at an acute angle to the glottis.[1] In anaesthetised patients, there is loss of tone in the submandibular muscles, tongue and indirectly the epiglottis. Flexible fibreoptic laryngoscope (FFL) is expensive, delicate, and the cables are not strong enough to lift or dislodge the tissues.[1] A number of fibreoptic-compatible oral airways (FCOAs) have been designed to protect the instrument from the patient’s teeth, guide it into the midline and keep the tongue from falling backwards.[2]

We have devised a modification of Guedel’s airway by removing the palatal surface of the airway [Figure 1]. The tracheal tube is threaded onto the fibrescope before laryngoscopy. The modified airway keeps the FFL tip around the back of the tongue, in the midline position and close to the larynx till it enters the trachea. The airway can be removed with a lateral twist movement without disturbing the endotracheal tube-endoscope assembly. Following this, the tracheal tube is advanced and the FFL is withdrawn.

**Figure 1:**
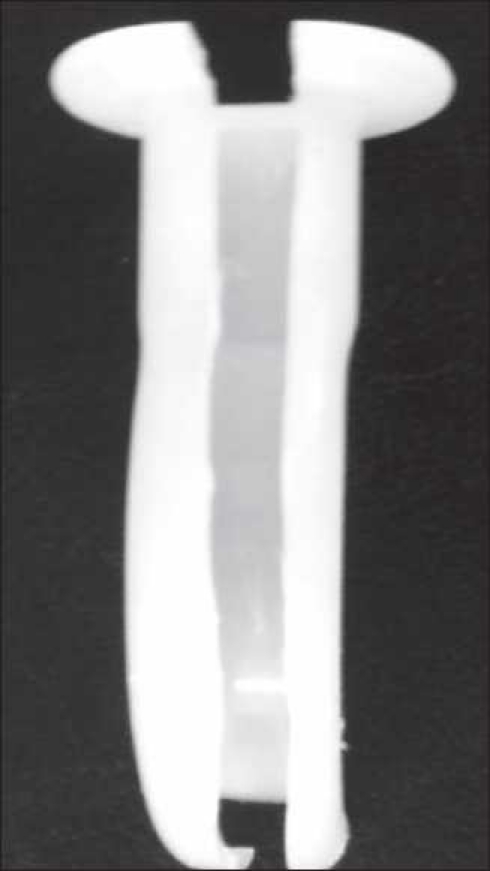
Guedel’s airway with slit on the palatal surface of the airway

On comparing it with anterior (lingual) channel like the Patil-Syracuse and the Williams airway intubator, the latter have the advantage of better localisation of the glottis opening but need to be removed from the oropharynx before a tracheal tube can be advanced over the fibre optic cable into the glottis, creating an unnecessary extra step.[1] McGinley and McAdoo[3] devised a modification of Guedel’s airway with deficiency in anterior support. However, at times, the tip of the FFL gets lodged in the pyriform fossa due to the deficiency in anterior support. Oral airways with a posterior channel, such as the Ovassapian and Berman, facilitate easy fibreoptic oro-tracheal intubation. Each can be rapidly removed from around the tracheal tube except for Burman’s airway as it has both posterior and lateral channels.[1] We have done a pilot study comparing both lingual and palatal modifications of Guedel’s airway and found that the palatal modification of Guedel’s airway makes FFLI easy, keeps the FFL in midline, no addition manoeuver like jaw thrust is required and causes no disturbance of endotracheal tube-laryngoscope assembly. A possible limitation of the modification would be lack of standardisation. Geudel’s airway is available in many sizes as compared to other FCOAs, making it versatile, cost-effective and highly acceptable.

## References

[1] Dorsch JA, Dorsch SE (2008). Understanding Anesthesia Equipment.

[2] Gal TJ, Miller RD (2005). Airway management. In: Miller’s anesthesia.

[3] McGinley J, McAdoo J (1999). Airway adjunct to an unanticipated difficult airway. Anesth Analg.

